# Gender Dysphoria in Adults: An Overview and Primer for Psychiatrists

**DOI:** 10.1089/trgh.2017.0053

**Published:** 2018-05-18

**Authors:** William Byne, Dan H. Karasic, Eli Coleman, A. Evan Eyler, Jeremy D. Kidd, Heino F.L. Meyer-Bahlburg, Richard R. Pleak, Jack Pula

**Affiliations:** ^1^ Mental Illness Research Education and Clinical Center, James J Peters VA Medical Center, Bronx, New York.; ^2^ Department of Psychiatry, Icahn School of Medicine at Mount Sinai and Center for Transgender Medicine and Surgery at Mount Sinai, New York, New York.; ^3^ Department of Psychiatry, University of California, San Francisco, San Francisco, California.; ^4^ Program in Human Sexuality, Department of Family Medicine and Community Health, University of Minnesota Medical School, Minneapolis, Minnesota.; ^5^ Departments of Psychiatry and Family Medicine, University of Vermont College of Medicine, Burlington, Vermont.; ^6^ Department of Psychiatry, Division on Substance Use Disorders, College of Physicians and Surgeons of Columbia University, New York, New York.; ^7^ Division of Gender, Sexuality, and Health, New York State Psychiatric Institute/Department of Psychiatry, College of Physicians and Surgeons of Columbia University, New York, New York.; ^8^ Department of Psychiatry, Division of Child and Adolescent Psychiatry, Hofstra North Shore-LIJ School of Medicine, Albert Einstein College of Medicine, Zucker Hillside Hospital, Ambulatory Care Pavilion, Glen Oaks, New York.; ^9^ Department of Psychiatry, Division of Gender, Sexuality and Health, College of Physicians and Surgeons of Columbia University, New York, New York.

**Keywords:** assessment, gender dysphoria, gender transition, mental health, psychiatry, intersex, transgender

## Abstract

Regardless of their area of specialization, adult psychiatrists are likely to encounter gender-variant patients; however, medical school curricula and psychiatric residency training programs devote little attention to their care. This article aims to assist adult psychiatrists who are not gender specialists in the delivery of respectful, clinically competent, and culturally attuned care to gender-variant patients, including those who identify as transgender or transsexual or meet criteria for the diagnosis of Gender Dysphoria (GD) as defined by *The Diagnostic and Statistical Manual of Mental Disorders* (5th edition). The article will also be helpful for other mental health professionals. The following areas are addressed: evolution of diagnostic nosology, epidemiology, gender development, and mental health assessment, differential diagnosis, treatment, and referral for gender-affirming somatic treatments of adults with GD.

## Introduction

Individuals who would likely be considered transgender today are evident throughout the historical record.^1^ The historical and sociocultural conceptualizations of gender variance, and their evolution within mental health professions over the past century and a half are reviewed elsewhere.^2^

Nineteenth and 20th century theories of gender variance and views of appropriate treatment were pathologizing and highly stigmatizing to transgender people.^2^ While mainstream psychiatry is now more affirming of gender variance, transgender individuals often are aware of the history in this area and many are likely to have encountered providers who adhere to outdated stigmatizing theories and approaches to treatment.^3^ Today's mental health professionals should, therefore, be familiar with the history in this area as it is not unusual for gender-variant patients to have apprehensions about seeking mental healthcare or to raise questions about their providers' views and approach to treatment considering that history.

Between 1963 and 1979, over 20 university-based gender identity clinics opened in the United States.^2,4^ These clinics provided interdisciplinary care that included psychiatrists and other mental health professionals and played an important role in the provision of medical services to transgender people and in promoting research to improve their care.^2,4^ The majority of these clinics closed following a 1981 decision of the U.S. Department of Health and Human Services (HHS) that labeled sex reassignment surgery as experimental,^5^ a decision what was overturned by HHS in 2014 in a determination that concluded that the 1981 decision was “unreasonable and contrary to contemporary science and medical standards of care.”^6^

With the closure of the academic gender clinics, transgender people in the United States came to rely on a loose network of medical and mental health providers, often affiliated with the Harry Benjamin International Gender Dysphoria Association (HBIGDA), which was subsequently renamed the World Professional Association for Transgender Health (WPATH). HBIGDA/WPATH developed and successively revised standards of care (SOC) for gender transition, which are currently in their seventh revision as the WPATH SOC7.^7^ In the WPATH SOC7, mental health professionals are tasked with determining whether those interested in gender-affirming treatments meet eligibility criteria, have capacity for informed consent, and have adequately anticipated the psychosocial impacts of their transition.

The WPATH SOC also provide clinical guidance for health professionals to assist transgender people in their search for psychological well-being in their gendered selves. In the absence of other comprehensive English language guidelines, U.S. providers and their professional associations came to rely heavily on the HBIDGA/WPATH SOC.^8–10^ Similarly, insurance carriers and tax courts employ WPATH SOC criteria in evaluating the medical necessity of transition treatments for determination of reimbursable and tax-deductible medical expenses.^11–14^

With transition services offered outside of university-based clinics, U.S. medical schools and residency training programs offered little exposure to the provision of transition services, leaving psychiatrists and other physicians poorly prepared for the growth in demand for these services seen in recent years.^15^ This article aims to assist adult psychiatrists and other mental health professionals who are not gender specialists in the care of these individuals. Detailed information on the assessment and treatment of gender dysphoria in children and adolescents can be found elsewhere.^16–19^

A glossary of transgender-related terms is found in Table 1. Providers should be respectful of their patients' identity labels; however, due to the rapid evolution of gender terminology, they may need to clarify how both their patients and colleagues employ particular terms.

**Table 1.** **Glossary**

Assigned gender: the initial gender attributed to an individual after birth; for most individuals, this corresponds to the sex on their original birth certificate, aka assigned gender, birth sex.^*^
Cisgender: a term for individuals whose experienced and expressed gender are congruent with their gender assigned at birth, that is, those who are not transgender.
Experienced gender: one's sense of belonging or not belonging to a particular gender, aka *gender identity*.
Expressed gender: how one expresses one's experienced gender.
Gender: a person's social status as male (boy/man) or female (girl/woman), or alternative category.^*^
Gender-affirming surgery: surgical procedures intended to alter a person's body to affirm their experienced gender identity, aka sex reassignment surgery, gender reassignment surgery, and gender-confirming surgery.
Gender assignment: assignment of a gender to an individual. In typically developed newborns, the initial gender assignment (aka “birth-assigned gender”) is usually made on the basis of the appearance of the external genitalia.
Gender binary: a gender-categorization system limited to the two options, male and female. Individuals who identify outside the gender binary may use a variety of gender identity labels, including genderqueer or nonbinary.
Gender dysphoria (not capitalized): distress caused by the discrepancy between one's experienced/expressed gender and one's assigned gender and/or primary or secondary sex characteristics.
Gender Dysphoria (GD) (capitalized): a diagnostic category in DSM-5, with specific diagnoses defined by age group-specific sets of criteria. This article addresses only GD in adults.
Gender identity: one's identity as belonging or not belonging to a particular gender, whether male, female, or a nonbinary alternative, aka experienced gender.
Gender Identity Disorder (GID) a diagnostic category in DSM-III and DSM-IV that was replaced in DSM-5 by *GD.*
Gender incongruence (not capitalized): incongruence between experienced/expressed gender and assigned gender, and/or psychical gender characteristics.
Gender Incongruence (capitalized): a diagnostic category (analogous to GD in DSM-5) proposed for ICD-11.
Gender role: cultural/societal definition of the roles of males and females (or of alternative genders).
Gender transition: the process through which individuals alter their gender expression and/or sex characteristics to align with their sense of gender identity.
Gender variance: any variation of experienced or expressed gender from socially ascribed norms within the gender binary.
Gendered behavior: behavior in which males and females differ on average.
Genderqueer: an identity label used by some individuals whose experienced and/or expressed gender does/do not conform to the male/female binary or who reject the gender binary.
Intersex conditions: a subset of the somatic conditions known as “disorders of sex development” or “differences of sex development “in which chromosomal sex is inconsistent with genital sex, or in which the genital or gonadal sex is not classifiable as either male or female. Some individuals who report their identity as “intersex” do not have a verifiable intersex condition.
Sex: a person's categorization as biologically male or female, usually on the basis of the genitals and reproductive tract.^*^
Sex assigned at birth: the sex or gender first assigned to an individual after birth. Also known as “natal gender,” “birth-assigned sex,” and “gender assigned at birth.” Often queried as “What sex was listed on your original birth certificate?”
Sexual orientation: a person's pattern of sexual attraction and physiological arousal to others of the same, other, both, or neither sex. Sexual orientation cannot be inferred from one's gender identity. As a show of respect, we recommend that the sexual orientation of transgender individuals be expressed in relation to their gender identity rather than their gender assigned at birth; however, all gender scholars do not follow that convention. Ambiguity in charting can be avoided by using terms such as sexually attracted to men, women, both, or neither.
^**^Transgender: an umbrella term usually referring to persons whose experienced or expressed gender does not conform to normative social expectations based on the gender they were assigned at birth.
^**^Transsexual: a term often reserved for the subset of transgender individuals who desire to modify, or have modified, their bodies through hormones or surgery to be more congruent with their experienced gender.

^*^ On official documents such as birth certificates, driver's licenses, and passports, the traditional category “sex” is equivalent to “gender” in current psychological terminology.

^**^ “Trans” (also “Trans^*^”) More recent umbrella terms being increasingly used to avoid distinguishing between transgender and transsexual individuals.

DSM, Diagnostic and Statistical Manual of Mental Disorders; GD, Gender Dysphoria; GID, Gender Identity Disorder; ICD, International Classification of Diseases.

## Diagnostic and Statistical Manual of Mental Disorders and Transgender-Related Nosology

The first two editions of the Diagnostic and Statistical Manual of Mental Disorders (DSM) published in 1952 and 1968, respectively, did not include any gender diagnosis.^20^ The diagnosis, “Transsexualism” (sic), first appeared in 1975 in the ninth revision of the International Classification of Diseases (ICD)-9^21^ and subsequently, in the DSM-III in 1980 under the parent category, Sexual Deviations.^22^ The defining characteristics of this diagnosis were as follows: (1) discomfort about one's assigned sex; (2) “cross-dressing,” in reality or fantasy, as the other sex, but not for the purpose of sexual excitement; and (3) the desire to get rid of one's primary and secondary sex characteristics and to acquire those of the other sex. DSM-III also included “Gender Identity Disorder of Childhood” (GIDC).

Both transsexualism and GIDC were carried over into DSM-IIIR, but were no longer categorized as sexual deviations. Instead, they were placed within the parent category, Disorders Usually First Evident in Infancy, Childhood, or Adolescence.^23^ This category also included disruptive behavior disorder, eating disorders, and tic disorders. Under this parent category, DSM-IIIR added a new diagnosis, Gender Identity Disorder of Adolescence and Adulthood Nontranssexual Type (GIDAANT). These changes recognized that gender identity disorder (GID) often begins in childhood, may or may not persist into adolescence and adulthood, and when it does persist, it may not entail a desire for the primary or secondary sexual characteristics of the other sex.

With DSM-IV, the diagnoses of Transsexualism and GIDAANT were discontinued, but GIDC and GIDAA were retained and placed under a new parent category, Sexual and Gender Identity Disorders, a category that also included the unrelated sexual dysfunctions and paraphilias.^24^ Individuals with somatic intersex conditions, who experienced dysphoria attributable to dissatisfaction with their gender assigned at birth, could be diagnosed with Gender Identity Disorder Not Otherwise Specified.

Retention of the diagnosis by the DSM and its new name, including the word “disorder,” was perceived by many as stigmatizing and contributing to societal discrimination against transgender individuals.^25^ By analogy to homosexuality, much of the distress and functional impairment associated with being transgender, and required for the diagnosis of GID, could derive from social stigmatization rather than from being transgender, *per se*. On the other hand, removal of a coded diagnosis for medical classification and billing purposes would limit access to transition care, deny the full impact of gender dysphoria, and prove harmful to transgender individuals.^2,26^

Ultimately, the diagnosis was retained by DSM-5,^27^ but its name was changed to Gender Dysphoria (GD), simultaneously removing the stigmatizing “disorder” from its name and shifting the focus to dysphoria as the target symptom for intervention and treatment, rather than gender identity itself.^27,28^ GD was also moved out of the parent category that included sexual dysfunctions and paraphilias, with which it has nothing in common, and into a separate parent category, also named Gender Dysphoria.

Use of the diagnostic label, GD, requires that a person meets the full criteria specified in DSM-5. This is distinctly different from the historical generic use of the term, gender dysphoria, which refers to the distress caused by a discrepancy between one's experienced gender and assigned gender, whether or not full DSM criteria for GD are met. For clarity here, references to the diagnosis will be capitalized or abbreviated (i.e., Gender Dysphoria or GD) while references to the symptom will not be capitalized or abbreviated (i.e., gender dysphoria).

The DSM is a manual on mental disorders and, therefore, despite the name change, GD retains its classification as a mental disorder. In contrast, the ICD is not limited to only mental disorders. In its forthcoming eleventh iteration, ICD-11, the diagnosis of Gender Incongruence (GI) (corresponding to GD in DSM-5 terminology) will most likely be moved out of the section on mental disorders. Instead, it has been proposed to place it in a separate section tentatively named Conditions Related to Sexual Health or Sexual and Gender Health.^29^ Placing GI in this section will declassify it as a mental disorder, while maintaining a diagnosis that will facilitate access to care through third party reimbursement, and could eventually lead to American Psychiatric Association (APA) removing GD from the DSM.

Importantly, the GD diagnosis does not apply automatically to people who identify as transgender but is given only to those who either exhibit clinically significant distress or impairment associated with a perceived incongruence between their experienced/expressed gender and their assigned gender or who, after transition, no longer meet full criteria, but require ongoing care (e.g., hormonal replacement therapy). In DSM-5, this latter group is given a “post-transition” specifier.

Unlike previous versions of the DSM, in DSM-5, gender-dysphoric individuals with somatic intersex conditions, who were previously excluded from the diagnosis, can now receive the diagnosis with a specifier to indicate the presence of the intersex condition. DSM-5 is also the first DSM to recognize the legitimacy of gender identities outside the gender binary such that individuals with GD are no longer described as identifying simply as “the other gender,” but as “the other gender (or some alternative gender different from one's assigned gender).” Examples of alternative genders include eunuch, genderqueer, and nonbinary.

## Epidemiology

Epidemiological research has employed different measures of transgender populations, resulting in varying estimates of prevalence.^30,31^ Some studies assessed the fraction of a population, which had received the DSM-IV diagnosis of GID or the ICD 10 diagnosis of transsexualism, both of which were limited to clinical populations who sought binary transition (male-to-female or female-to-male). For example, the prevalences reported in DSM-5 (0.005–0.014% for birth-assigned males; 0.002–0.003% for birth-assigned females) are based on people who received a diagnosis of GID or transsexualism, and were seeking hormone treatment and surgery from gender specialty clinics,^25^ and, therefore, do not reflect the prevalence of all individuals with gender dysphoria or who identify as transgender.

The prevalence of transgender people receiving gender specialty care in the Netherlands has been estimated at 0.008% for transgender women and 0.003% for transgender men.^32^ More recent data for those obtaining surgery in Belgium were similar.^33^ In Sweden, point prevalence in 2010 was estimated to be 0.013% for transgender women and 0.008% for transgender men.^34^ A higher percentage, 0.023%, received a diagnosis of GID recorded in the health records of the U.S. Veteran's Administration.^35^

Other studies, rather than measuring the proportion of a population that received a clinical diagnosis, have reported on those who self-identified as transgender or gender incongruent, and found that measuring self-identity yields much higher numbers. In 2016, data from the Center for Disease Control's Behavioral Risk Factor Surveillance System suggested that 0.6% of U.S. adults identify as transgender, double the estimate utilizing data from the previous decade.^36^

In a large Massachusetts population-based phone survey, 0.5% of the population (age 18–64 years) identified as transgender.^37^ In another large population-based survey in the Netherlands, 1.1% of those assigned male at birth (age 15–70 years) reported an incongruent gender identity (stronger identification with a gender other than the one assigned at birth), as did 0.8% of those assigned female at birth.^38^

Recent surveys of youth showed even higher numbers. In New Zealand, 1.2% of high school students surveyed identified as transgender.^39^ In a survey of San Francisco middle school students (grades 6–8), 1.3% identified as transgender.^40^ More study is needed, but these larger numbers indicate that many transgender people have not been counted in clinical studies, including those with nonbinary identities, those not seeking transition care, those receiving hormones outside of clinics specializing in transgender care or by self-administration, and others who identify as transgender when surveyed, but do not report gender dysphoria to clinicians.

## Gender Development

### Biological considerations

Animal research has established that sex differences in the phenotype of both body and brain as well as behaviors are the result of multiple, sex-biasing factors. These include hormonal, sex-chromosomal,^41^ genetic, and epigenetic contributions.^42^ The sensitivity of brain tissues to organizational effects of sex hormones appears to be particularly high at prenatal/perinatal stages of development and gradually declines toward young adulthood.^43^ The timing of hormonal secretions in the course of development, however, gives the impression of three discrete sensitive periods: (1) pre/perinatal; (2) pubertal^44^; and (3) for females, the first pregnancy.^45^

In humans, statistical sex differences in brain structure are well documented,^46^ and findings of sensitive periods for sexual differentiation of the brain appear to parallel those seen in other mammals.^47,48^ The evidence for brain/behavior effects of prenatal androgenization is particularly strong,^49–51^ much of which derives from studies of individuals with somatic intersex conditions and varying degrees of functional androgen exposure.^51–53^

Androgenization of the brain depends not only upon the level of androgen to which a fetus is exposed but also upon numerous other factors, including the presence of enzymes to convert androgens to the specific metabolites required by particular brain cells, their steroid receptors, and their postreceptor mechanisms that are involved in the full response to androgens. Receptor structure, which can influence sensitivity, is genetically determined, while the activity of genes for receptors and postreceptor mediators is subject to epigenetic modulation.^54^

As the period of genital differentiation largely precedes the sexual differentiation of the brain,^55^ it is conceivable that GD in individuals without somatic intersex conditions could reflect a brain-limited intersex condition (i.e., a lack of concordance between the sexually differentiated state of the brain and body). That hypothesis has been tested in a variety of ways, including searching for features of the brain in individuals with GD that more closely match their experienced gender than their birth-assigned gender.^56^ Investigations in this regard have included postmortem morphometric and stereological studies,^57^ as well as *in vivo* morphometric,^58^ functional magnetic resonance imaging,^59^ and diffusion tensor imaging studies of the brain,^60–62^ and examination of otoacoustic emissions.^63^

As reviewed elsewhere,^53,56,64^ while some positive findings in the predicted direction have been reported,^56,64^ inferences are currently limited. This is because few findings have been replicated and few studies have adequately controlled for potentially confounding variables such as age, sexual orientation, transition status (including history of gender-affirming hormonal treatment, if any), and hormonal status at the time of study (or of death in the case of postmortem studies).^53^

Much of what is known about the role of early hormonal exposure on the development of gender identity in humans derives from studies of gender outcomes in individuals with somatic intersex conditions. Early guidelines for initial gender assignment for such infants relied heavily on the surgical potential to achieve concordance between the gender assigned and the appearance and functional potential of the external genitalia, in particular, the capacity of penile-vaginal intercourse.^9^ Current guidelines, however, emphasize what is known about the long-term gender outcomes of individuals with intersex conditions on a syndrome by syndrome basis.^52^

Overall, these data suggest that regardless of genetic constitution, or gonadal or genital development at birth, individuals prenatally exposed to a full complement of masculinizing hormonal influences (i.e., androgen exposure and the cellular mechanisms for responding fully to androgens as described above) have an increased likelihood of GD when assigned female.^51,52^ Conversely, most reported 46,XY individuals with complete androgen insensitivity syndrome (and hence no functional androgenization of the brain) have developed a female gender identity, despite having a Y chromosome as well as normally developed and functioning testes.^51,52^ To date, however, no brain marker of sexual differentiation has been validated to guide the initial gender assignment of infants with intersex conditions.

### Psychosocial factors influencing gender expression

In mammals, and particularly in humans, psychological and social factors have a major additional influence on behavioral outcome.^65^ In humans, these psychosocial processes include verbal labeling (e.g., “boy” and “girl”) and nonverbal gender-cuing (e.g., gender-specific clothing and haircuts) of children by parents and others in their social environment, as well as the shaping of children's gendered behavior by positive and negative reinforcement and later by explicit statements of gender-role expectations. Related processes in developing children include gender-selective observational learning/imitation, the formation of gender stereotypes and of related self-concepts, and self-socialization. The effects on gender development have been documented in a vast body of research in developmental psychology.^65^

The impact of such psychosocial factors, however, is not determinative. This is evidenced by individuals in whom gender identity is discordant with the initial gender assignment and gender of rearing, for example, transgender individuals and a higher than expected proportion of individuals with particular intersex conditions (i.e., 46,XY individuals with high degrees of somatic hypomasculinization and 46,XX individuals with high degrees of somatic hypermasculinization^66,67^).

### Factors in gender-identity development

Systematic data on gender identity development are much more limited than those on gendered behavior. Yet, the data available, especially for those with intersex conditions, lead to the conclusion that, while early androgenization plays a role, a definitive biological predetermination of gender identity seems unlikely. Not a single biological factor, but multiple factors (i.e., biological, psychological, and social) appear to influence the development of gender identity.^50^

The need to transition gender is even less understood in individuals without, compared to those with, intersex conditions.^68^ Along with the dramatically increased referrals of gender-variant individuals to specialized clinics in Western Europe and North America over the last two decades,^69,70^ there has been a diversification of presentations beyond the original “transsexual” who sought (or was perceived by providers to seek) change to the “other” gender through treatment with gender-affirming hormones and genital surgeries. Currently, many transgender people seek chest, but not genital surgery, or only gender-affirming hormones, or only a social transition without any medical changes. Others may simply desire flexibility in gender expression without transition to “the other gender,” identifying, for example, as nonbinary or genderqueer.^71,72^

Prospective follow-up studies of children, who before puberty had met criteria for the DSM-IV diagnosis of GID, showed that the majority of those diagnosed with GID in early or early middle childhood “desisted,” meaning that they subsequently identified as their birth-assigned gender and did not meet criteria for GID. As adults, many identified as lesbian, gay, or bisexual.^73–75^ Some “desisters,” however, subsequently transitioned later in life.^73^

The data available do not allow a clear prediction before puberty of which child will persist and transition permanently, and which child will not.^75^ With the introduction of stricter criteria for the diagnostic category of Gender Dysphoria in DSM-5, the persistence rate likely will be higher,^73^ but this needs to be tested by future long-term follow-up studies. For example, the degree of gender nonconformity and whether a child believes they *are*, as opposed to *wishes to be*, “the other” gender have been proposed as predictors of persistence.^76,77^ Those in whom GD persists from childhood into adolescence are likely to experience an exacerbation of dysphoria with the emergence of (or with the anticipation of) undesired secondary sexual characteristics at puberty, in which case pubertal suspension should be considered.^10^

Regardless of their initial sexual orientation, during and after transitioning to express their experienced gender, some individuals retain their pretransition sexual attraction patterns, while others change.^7^ In some transgender women, the desire to transition gender is preceded by fantasizing themselves as women, sometimes with sexual arousal.^78^ This phenomenon has been controversially interpreted by some as fetishism.^79^ Importantly, neither a history of fetishistic arousal nor one's sexual orientation precludes one from meeting the criteria for the diagnosis of GD^27^ or eligibility for gender transition services.^7,80^

## Mental Health Assessment and Treatment

This section addresses the assessment and treatment of adults with gender identity or expression concerns in the absence of an intersex condition. GD in individuals with intersex conditions is addressed in the Appendix. Treatment of GD in prepubescent children, where there is currently less consensus,^81^ is addressed elsewhere as is treatment of adolescents, including selection of candidates for pubertal suspension.^81,82^ The primary roles of the mental health professional in assessing and treating patients with GD are based on expert consensus,^7,8,10,20^ summarized in Table 2 and described more fully below in the broader context of gender variance.

**Table 2.** **Roles of the Psychiatrist**

Assess and diagnose gender concerns according to current DSM criteria and see that they are addressed.
Assess and diagnose any coexisting psychopathology and see that it is addressed.
Assess eligibility for hormonal and/or surgical treatments, or refer to professionals capable of making such assessments.
Assess capacity to give informed consent for hormonal and surgical treatments.
Ensure that eligible individuals are aware of the full range of treatment options and their physical, psychological, and social implications, including risks, benefits, and impact on sexual functioning and reproductive potential.
Ensure adequate psychological and social preparation for transition treatments.
Refer patients for hormonal or surgical treatments, collaborating with providers as needed.
Ensure continuity of mental healthcare as indicated throughout transition and beyond.

Expert consensus regarding the treatment of adults has been arrived at after many years of clinical experience. Attempts to engage individuals in psychotherapy to change their gender identity or expression are currently not considered fruitful by the mental health professionals with the most experience working in this area^7,9,83^ and legal bans of therapies aimed at changing sexual orientation have recently been extended to therapies aimed at changing gender identity or expression in a number of U.S. states and Canadian provinces.^84,85^ Currently, psychotherapeutic involvement with adults with GD is primarily used to assist in clarifying their desire for, and commitment to, changes in gender expression and/or somatic treatments to minimize discordance with their experienced gender, and to ensure that they are aware of and have considered alternatives.^7^

Gender questioning, gender-variant, and transgender adults present to mental health services for a variety of reasons. Some presentations may relate explicitly to gender. For example, patients may wish to explore their gender identity, consider transition options and concerns (e.g., coming out to family or coworkers), or request evaluation for hormonal or surgical treatments. The latter may include requests for referrals for such treatments, including requests for mental health referral letters as specified by the WPATH SOC7 or required by their providers of transition treatments and/or insurance carriers.^7,11–13^

According to WPATH SOC7, as an alternative to an evaluation by a mental health professional, primary care providers who are competent in the assessment of GD may evaluate patients for hormone therapy, particularly in the absence of significant coexisting mental health concerns and when working in the context of a multidisciplinary specialty team.^7^

Patients may also seek couples or family therapy before, during, or after transition to address the impact of the transition on interpersonal or family dynamics. Alternatively, many transgender patients seek or are referred to psychiatric services for reasons that are either unrelated to gender identity or expression (e.g., management of primary psychiatric illnesses), or only partially related (e.g., sequela of childhood trauma as a result of minority stress due to gender nonconformity).

A careful evaluation for a history and psychological sequela of gender-related stigma and abuse, from childhood on, is crucial given the high rates of violence and bullying experienced by gender-variant individuals, as well as the high rates of discrimination, unemployment, homelessness, sex work, and HIV infection.^3,86^ High rates of depressive, anxiety, and substance use disorders, as well as suicidal ideation and completed suicide have been linked to such gender minority stress.^87–89^ In addition to these mental health disparities, the transgender population also exhibits marked general health disparities.^90^ Few of these disparities are linked to sexually transmitted infections or hormonal or surgical transition treatments,^7,10,90^ but are instead linked to financial barriers to care as well as avoidance of healthcare due to experienced and/or anticipated stigma and discrimination in healthcare settings, and the widespread belief among transgender individuals that medical professionals are poorly trained to meet their needs,^3^ a belief that appears to be well-founded.^15^ Extensive guidance on overcoming these barriers to care, including creating a welcoming clinical environment, can be found elsewhere.^91^

### Assessment of gender concerns

Treatment should be patient centered and tailored to the needs and individuality of each patient. Patients should be asked what names and pronouns they use and should be addressed by those names and pronouns regardless of their stage of transition. Those who transitioned many years ago and are seeking treatment for another problem typically need much less focus on gender history than those who are questioning their gender identity, just beginning gender transition, or exploring options for gender expression. When gender is not the primary concern, devoting the appropriate amount of attention to gender-related issues is important, balancing against an overemphasis on gender that can feel inadvertently stigmatizing to the patient or distract from adequate focus on the chief complaint.

While it is important to avoid the assumption that coexisting psychiatric symptoms are due to gender variance, the impact of past and present gender-related stigma should be considered in the biopsychosocial evaluation. This is particularly important in light of the stress-diathesis model of psychiatric illness and its exacerbations.^8,92^ Suicidality should always be assessed, as should protective factors such as social and family supports.^93^ Suicidal ideation^3,94^ and completed suicide^90^ are dramatically increased in this population and GD may be a risk factor for suicidality, independent of other psychiatric conditions.^94,95^ Up to 47% of transgender adults have considered or attempted suicide.^93^ Assessment of suicide risk is especially important during periods of heightened vulnerability, such as when transgender identity is disclosed to family and more broadly.^9,83^

The gender assessment should include the age and circumstances when the patient first became aware of a sense of difference from peers of the same sex assigned at birth as well as experiences of negative affect or self-perception related to that sense of difference.^8,20^ Any history of peripubertal and/or pubertal distress due to the anticipation and/or emergence of unwanted secondary sex characteristics should also be explored, as should past experiences of gender-related stigmatization, discrimination, harassment, and violence.^8,20^

The patient's history of coping mechanisms and support systems should also be examined.^8,20^ Gender expression (e.g., pronoun use, name changes, manner of dress, and bodily modifications) over time should be explored as well as what has and has not been helpful in improving the sense of well-being. It is important to clarify each patient's goals and plans for social and/or medical transition, degree of commitment, and expectations.^7,96^ For those who do not wish to transition, assessing current psychosocial challenges and formulating with the patient how to best address them (e.g., psychotherapy, group therapy, and social support) should not be neglected.

Recommendations regarding psychiatric assessment of individuals with GD have focused largely on assessment of eligibility for and decision-making capacity related to medical and surgical gender transition services.^7,8,10^ Eligibility for both gender-affirming hormone therapy and surgeries requires persistent gender dysphoria, a documented diagnosis of GD based on DSM-5 criteria, and the capacity to give informed consent.^7^ In addition, any significant medical or psychiatric concerns must be sufficiently controlled so that they do not interfere with the patient's ability to safely adhere to the treatment regimen. The current standard of care in major clinics, the WPATH SOC7, and insurance requirements for reimbursement of services follow a flexible progression of transition steps, which may begin with completely reversible steps (e.g., change of pronouns, name, and manner of dress), followed by partially reversible changes (e.g., gender-affirming hormones), and then irreversible gender-affirming surgeries.^7,10–14,97^ There is flexibility in this process given that some people do not pursue all of these interventions or may prefer to do so in a different sequence. For example, transgender men may wish to undergo mastectomy or male breast construction before initiating masculinizing hormones.^7^

Before gonadectomy, 12 months of continuous hormone therapy consistent with the patient's gender goals are recommended, unless hormones are clinically contraindicated for the individual. The aim of hormone therapy before gonadectomy is primarily to allow the individual to experience a period of gender-affirming hormones, before irreversible surgical intervention.^7^ Before masculinizing or feminizing genital reconstructive surgeries, the WPATH SOC7 also recommend 12 continuous months of living in a gender role that is congruent with the patient's gender identity.^7^

#### Diagnosis of gender dysphoria

The DSM-5 diagnostic criteria for GD in adolescents and adults are shown in Table 3. Diagnosing GD in adults by these criteria is usually straightforward, especially for those with overt manifestations in childhood, exacerbation of distress with pubertal changes, and persistence into adulthood in the absence of significant coexisting mental health concerns.^8,9^

**Table 3.** **Diagnostic Criteria for Gender Dysphoria in Adolescents and Adults**

A marked incongruence between an individual's experienced/expressed gender and assigned sex as evidenced by two of the below, which have been present after the onset of puberty for at least 6 months:
A marked incongruence between one's experienced/expressed gender and primary and/or secondary sex characteristics (or the anticipated secondary sex characteristics in young adolescents).
A strong desire to be rid of one's primary and/or secondary sex characteristics because of a marked incongruence with one's experienced/expressed gender (or a desire to prevent the development of the anticipated secondary sex characteristics in young adolescents).
A strong desire for the primary and/or secondary sex characteristics of another gender.
A strong desire to be of a gender different from one's assigned gender.
A strong desire to be treated as a gender different from one's assigned gender.
A strong conviction that one has the typical feelings and reactions of a gender different from one's assigned gender.
The condition is associated with distress or impairment in social, occupational, or other important areas of functioning that are clinically significant.

Adapted from DSM-5.^27^

Assessment of patients who are seeking transition services, but do not clearly meet criteria for GD, may require more time and exploratory therapy^9^ (e.g., a patient desiring hormonal or surgical treatment to transition to another gender, who does not clearly experience incongruence between their experienced gender and their gender assigned at birth). The same is true for those with the onset of gender dysphoria in the context of a psychiatric disturbance (e.g., psychosis, dissociative disorder, and autism spectrum disorder) or recent trauma^9,98,99^; those who are ambivalent about their gender identity or desired sex characteristics; and those who exhibit marked exacerbations and remissions of dysphoria over time.

The psychiatrist must assess whether some factor other than GD accounts for the expressed desire to transition. If not, coexisting mental illness is not a contraindication to supporting transition if it is sufficiently controlled to not interfere with the patient's capacity for decision-making or ability to safely adhere to the demands of the desired treatment.^7,9,98^

#### Differential diagnosis

Few conditions can be mistaken for GD. Simple nonconformity to gender roles can be differentiated from GD based on the degree of associated distress and whether or not the individual identifies as the sex assigned to them at birth. GD can be differentiated from body dysmorphic disorder (BDD), in which an individual may wish a body part to be removed or altered because it is viewed as deformed.^27^ In contrast, in GD alterations are sought for anatomical characteristics that are incongruent with one's gender identity. BDD and GD can, however, coexist and the presence of BDD is not an absolute contraindication for gender-confirming surgery.^27^ Transvestic disorder is characterized by significant distress or impairment due to sexual arousal in the context of cross-dressing fantasies, urges, or behavior. It may exist independently or co-occur with GD,^27^ and is not a contraindication to supporting transition in those who meet criteria for GD.^7^

Gender-themed delusions have been reported to occur in up to 20% of those with psychotic disorders.^100^ Such delusions can usually be easily differentiated from GD by their content (i.e., if they do not entail the belief that one's gender differs from that assigned at birth), as well as by their presence only during psychotic phases of illness, and the absence of other DSM criteria required for the diagnosis of GD.^98^ Importantly, GD and psychotic disorders may coexist and patients with both diagnoses can benefit from gender-affirmative treatment and appropriate hormonal and/or surgical gender interventions.^98^ Timely diagnosis of GD may be impeded when it is first overtly expressed in adolescence or early adulthood coincident with, or shortly following, the first psychotic episode.^98^

### Mental health treatment

Statements in this section are based on the cited studies supplemented by the authors' cumulative clinical experience treating patients with GD. Psychotherapy can be useful for patients with GD; however, many successfully transition or decide against transition with little or no psychotherapy. Psychotherapy may be helpful at different times and for different reasons across the lifespan.^7^ Many transgender people seek mental health treatment on an intermittent basis, while contemplating gender transition, at key points in the transition process, or post-transition if symptoms recur or worsen.

Participation in transgender support groups, including peer-led groups, and other interactions with transgender individuals or the transgender community are often useful in clarifying the goals of those who experience ambivalence about transition. With patients who are otherwise eligible for transition treatments, but express ambivalence about transition, the therapist should maintain a stance of neutrality, creating a safe therapeutic space in which the patient can weigh all options and arrive at a decision in their own time. Many transgender adults need some combination of hormonal treatment and/or surgical procedures for relief of GD, but some experience relief with a change in gender expression without any medical treatment.^7^ Strengthening resilience factors identified in the transgender population^93^ should be a focus, particularly, in patients with suicidal ideation.

Although treatment with exogenous estrogen or testosterone carries a risk for medical side effects,^10^ both have been associated with improvement with respect to anxiety, mood, and mood stability, as well as overall satisfaction and quality of life for both transgender women and transgender men.^101–104^ Similarly, review of the available literature^9^ demonstrates the benefits of surgery in alleviating GD and the rarity of postsurgical regret. Emotional changes may occur with use of either androgen or estrogen supplementation, although these are usually subtle.^9^ An increase in libido usually occurs with androgen use with female to male transition.^10^ Although decreased libido due to antiandrogen and/or estrogen treatment in individuals transitioning male to female is common,^10^ some may experience a stronger interest in sex, perhaps due to the affirming aspects of attaining desired bodily changes.

Safer sex information and instruction in self-protective negotiation in sexual settings should be provided and tailored to the anatomy, needs, and experiences of transgender persons.^9^ Masculinizing hormones have been associated with a possible destabilization of psychotic and bipolar disorders, especially with supraphysiological blood levels of testosterone^7^ in both cisgender and transgender men.^105–106^ The likelihood of such episodes can, therefore, be minimized by careful dosing and monitoring.

Detailed information on specific gender-affirming surgical procedures can be found elsewhere.^7,107^ Psychiatrists should collaborate with other providers (e.g., endocrinologists, surgeons, psychotherapists, primary care providers, social workers, and other mental health professionals) to ensure that patients have the knowledge required to adequately evaluate the benefits, risks, and limitations of desired treatments and their alternatives. This is necessary not only for informed consent but also to ensure adequate preparation for surgery and postsurgical needs (e.g., convalescent period, period of sexual abstinence, and vaginal dilatation in the case of vaginoplasty).

Helping the patient anticipate and prepare for psychosocial impacts of treatment (e.g., impact on social relationships and employment) is also essential. Importantly, transition treatments target GD, not coexisting psychiatric diagnoses, and coexisting diagnoses are likely to require ongoing attention after transition, although symptom severity may be ameliorated.^98,100,102^

### Referrals for hormones and surgery

Whether the initial evaluation for hormones is done by the hormone prescriber or by a mental health professional, criteria for starting hormones are the same: the presence of persistent GD, the ability to give informed consent, and relative mental health stability.^7^ Insurance carriers and surgeons require mental health evaluation before transition-related surgeries to assess and document eligibility, readiness, and medical necessity of the requested procedure.^7,10–14^

The specific requested content of referral letters varies among surgical providers and insurance plans. To avoid unnecessary delays in treatment, letter writers should be aware of such differences and ensure that their letters meet the requirements of all relevant parties. The content requested by most providers and insurance carriers is similar to that outlined in the WPATH SOC7. Genital and gonadal surgeries usually require documentation from two licensed mental health professionals, while chest surgeries generally require just one evaluation and referral.^7,108^ Although not requirements of WPATH SOC7, some insurers require one letter from a psychiatrist or other doctoral level mental health provider, or may specify a minimal duration of mental healthcare.^13^ Such requirements vary by health system, insurance carrier, and state, and raise challenges for those without access to reimbursement for mental healthcare.

## Current Social Issues: Stigmatization and Access to Care

Transgender health advocates have worked to address societal discrimination against transgender people, including stigmatization of identity, discrimination in schools, workplaces, and healthcare, and to improve access to care. Increasingly, this advocacy has been embraced by major institutional and governmental agencies. One large online survey, the National Transgender Discrimination Survey^88^ found that rejection, discrimination, victimization, and violence against transgender people occur in a multitude of settings and negatively affect transgender people across the life span. Transgender youth are often harassed and assaulted in schools, which is associated with dropping out and subsequent impoverishment. Many transgender people are harassed at work or lose jobs due to their gender identity and expression. Discrimination extends to healthcare settings, where patients may be refused care or treated disrespectfully, or do not have access to care.^88^

U.S. public policy has contributed to the lack of access to care. A report by the National Center for Health Care Technology of the HHS Public Health Service issued in 1981, titled “Evaluation of Transsexual Surgery,” deemed these procedures “experimental,” and recommended that Medicare not cover transition-related care. This was formalized in a 1989 Health Care Financing Administration National Coverage Determination.^5^ Exclusion of transgender healthcare in private insurance as well as Medicaid and Medicare was near universal in the decades to come. A lack of funding for clinical care and research led to the closing of transgender care programs at academic institutions in the years following the 1981 report.

Many transgender health insurance exclusions have been removed recently. This trend started with increasing numbers of employers in the last 15 years adding transition care to health coverage. Starting in 2013, some states have ruled that transgender healthcare exclusions are discriminatory and have banned them from state-regulated health insurance plans. In 2014, the 1981 Medicare policy was reversed, removing categorical exclusions for transgender care.^6^ In 2015, the HHS moved to end categorical exclusions for transgender care from all insurance and care providers who accept federal funding or reimbursement;^109^ and since 2016, insurers in the Federal Employees Health Benefits Program must include transition-related coverage for transgender federal employees.^110^

During this same period, executive orders and other guidance from the Obama administration conferred increased protection against discrimination to transgender individuals in workplace and educational settings,^111^ the ban on open military service of transgender individuals was lifted,^112^ and changes at the HHS and the National Institutes of Health (NIH) facilitated research to better define and address the health needs of transgender individuals.^111^ Much work remains, however, to fully actualize these policy changes. In addition, progress has been slowed on the federal level by the change in presidential administrations and legal actions.^113^

WPATH SOC7^7^ has attempted to improve access to care by including the informed consent model for hormone administration. In multidisciplinary clinics providing transgender care, primary care providers can assess for and diagnose longstanding GD that might benefit from treatment with hormones and administer hormones without referral from a mental health professional. However, patients with cooccurring mental health conditions should be referred to mental health providers when appropriate. WPATH has advocated for the depathologization of transgender identity, the medical necessity of transgender care, and improved access to legal gender change.^7^

The APA has also attempted to reduce stigma and improve access to care. As discussed previously, the DSM-IV diagnosis of GID, regarded as stigmatizing by many transgender health and advocacy groups, was replaced with GD in DSM-5.^114^ In addition, the APA approved position articles on discrimination and access to care. Its statement on discrimination against transgender and gender-variant individuals^115^ opposes all private and public discrimination against transgender individuals, and its statement on access to care for transgender and gender-variant individuals^116^ urged the removal of all categorical healthcare exclusions for transgender people and advocated for the expansion of access to care.

Increased access to care must be accompanied by culturally competent research in transgender health, recommended by the Institute of Medicine^86^ and outlined in the NIH's Strategic Plan to Advance Research on the Health and Well-being of Sexual and Gender Minorities.^117^ Expanded and improved education of healthcare providers is necessary, and the American Association of Medical Colleges has produced guidelines for curricular and climate change to improve transgender health.^118^ Principles of culturally competent care for transgender and nonbinary patients should be included in residency training as well, including psychiatric residency programs.

## Conclusions

Transgender, nonbinary, and gender questioning people are sufficiently common that even psychiatrists whose practice does not focus on gender are likely to encounter patients who have transitioned gender, are planning or considering transition, or are questioning their gender identity. Gender concerns are only one of the reasons these individuals may seek psychiatric care and, regardless of their area of specialization, psychiatrists should be adept at conducting respectful, culturally sensitive, and affirming gender assessments without placing an undue emphasis on gender when it is not the patient's presenting concern. Mental health professionals must fully appreciate that the focus of treatment for GD is on the dysphoria, not the gender identity. At the same time, they must appreciate the role of minority stress in gender minority mental health disparities, screen for related manifestations, including anxiety disorders, depression, and suicidality, and consider resilience factors in treatment planning.

Psychiatrists should also be competent in the provision of routine psychiatric care that is gender affirming to gender variant patients with serious mental illnesses without assuming that the gender variance is a manifestation of the illness. They should not expect coexisting serious mental illness, especially in the context of strong genetic loading, to fully resolve with successful treatment of GD and should assist the patient in formulating realistic expectations.

If not included in their residency or fellowship training, or supervised clinical experience, psychiatrists should familiarize themselves with the standards of care for gender transition as described in the WPATH SOC7 and outlined in this article, as well as the roles and minimal competencies of mental health professionals working with adults with GD.^7^ In addition to the minimal competencies, WAPTH SOC7 recommends that health professionals take steps to sustain or augment their cultural competency to work with transgender and other gender minority patients by participating in continuing education and becoming knowledgeable about community, advocacy, and public policy issues that affect transgender individuals and their families.^7^

All providers should work within their sphere of competency and refer patients when necessary. Board-certified psychiatrists should be competent in the diagnosis of GD by the criteria of the most current DSM and in assuring that any coexisting psychiatric disorder is appropriately diagnosed and adequately controlled.^118^ In the absence of additional training, they should refer to other providers or seek supervision in fulfilling the other tasks of mental health professionals in addressing the gender concerns of transgender and other gender diverse patients. Providers from all disciplines should work within their professional organizations to ensure that training in gender-affirmative care is integrated throughout all levels of the training curriculum.^119^

## Abbreviations Used

APA: American Psychiatric Association
BDD: Body Dysmorphic Disorder
DSM: Diagnostic and Statistical Manual of Mental Disorders
GD: Gender Dysphoria
GID: Gender Identity Disorder
GIDAANT: Gender Identity Disorder of Adolescence and Adulthood Nontranssexual Type
GIDC: Gender Identity Disorder of Childhood
HBIGDA: Harry Benjamin International Gender Dysphoria Association
HHS: U.S. Department of Health and Human Services
ICD: International Classification of Diseases
NIH: National Institutes of Health
SOC: Standards of Care
WPATH: World Professional Association for Transgender Health

## Appendix

### Gender Dysphoria in Patients with Intersex Conditions

As reviewed elsewhere,^A1^ Gender Dysphoria (GD) and patient-initiated gender transition occur with increased frequency in individuals with intersex conditions. Because Diagnostic and Statistical Manual of Mental Disorders-5 now allows gender-dysphoric individuals with somatic intersex conditions to receive the diagnosis of GD, psychiatrists need to be aware of assessment- and treatment-relevant characteristics of such individuals that differ from gender-dysphoric individuals without somatic intersexuality.^A2^

Intersex conditions are a subset of conditions relatively recently designated as “disorders of sex development”^A3^ or “differences of sex development” (DSD).^A4^ We use the term “intersex” in this document as our focus is on that subset of individuals with DSDs who were born with atypical external genitalia or lack of concordance among various sex characteristics such as sex chromosomes, gonads, or external genitalia so that questions often arise as to which gender should be assigned at birth. GD may develop from late preschool age through late adulthood with a range from 0% to ∼70% depending on the specific intersex syndrome, its severity (degree of androgen insensitivity, degree of 21-hydroxylase deficiency, degree of genital atypicality, etc.), the gender originally assigned, and the postnatal history of exposure to both endogenous and exogenous sex hormones.^A5^

Persons with the combination of GD and intersex condition encounter fewer barriers to legal gender reassignment, and the barriers to hormonal and surgical treatments are much lower.^A1^ This is because, depending on the particular condition, individuals with an intersex condition may have been gonadectomized (often due to concern about risk of malignancy) before puberty so that administration of exogenous hormones is required as part of routine care to induce puberty. In addition, infertility is quite common whether due to the condition itself or to gonadectomy, and genital surgery has often been done in infancy or childhood with the intent of affirming, both to the patient and the parents, the gender to which the individual was assigned. Furthermore, such early procedures may have been followed by additional surgical modifications in adolescence or young adulthood.

Decisions regarding hormonal and surgical procedures are complicated by the highly variable somatic presentations of the various intersex conditions. Thus, to be fully effective, the mental health provider needs to be informed about the medical and surgical history of the individual,^A6,A7^ the available data on long-term gender development (e.g., contentment vs. dysphoria in the assigned gender), and other psychological outcomes of patients on a syndrome by syndrome basis.^A5,A8^ Moreover, intersex conditions are frequently associated with stigma, even in medical settings, which may result in shame and maladaptive coping mechanisms on the part of the patients as well as their parents.^A9–A12^

Providers need to be aware of the many ways in which some individuals with intersex conditions report having been stigmatized by their treatment by clinicians and parents (e.g., failure of age-appropriate disclosure of their condition, attempts to modify their gender expression, and repeated genital examinations^A9,A13^). Efforts are under way to develop decision-making tools and clinical checklists to ensure that parents and affected children are adequately assessed and informed as active participants in decision-making processes and that the intersex condition and its ramifications are disclosed to the affected individual in an age-appropriate manner.^A14^

### Gender evaluation

The questionnaires and interview schedules developed for the assessment of gender development in transgender individuals who do not have an intersex condition^A15,A16^ apply to those with intersex conditions as well, but need to be complemented by detailed medical, surgical, and related psychosocial histories, including the histories of disclosure to the patient of her/his medical condition, efforts made to reinforce the initial gender assignment, and responses by parents and providers to behaviors perceived as atypical with respect to the gender assignment. Mental health providers should also assess the patient's knowledge of their surgical history, their understanding of the implications with respect to fertility and gender-affirming hormonal and surgical procedures, and any history of shaming or other stigma due to their condition, or perceived gender atypicality with respect to their gender assigned at birth.

### Decisions regarding gender transition

For individuals with intersex conditions, GD usually raises the question of transition to a different gender, and all issues of relevance to transgender persons without these conditions should also be considered here. Yet, the situation is often more complex than in GD in the absence of an intersex condition. Factors contributing to the desire to transition may include the awareness of the discrepancy between assigned gender and genetic factors such as the karyotype, anatomic factors such as the type of gonads, and secondary sex characteristics like breast development in men or hirsutism and masculine habitus in women. Related psychosocial influences may derive from being misidentified as the “other” gender or from frank stigmatization due to gender-atypical physical features.

Different cultures and even subcultures within a given country may differ in the roles (including rights) associated with one's gender, and in the salience and weight of criteria used in decision-making on gender reassignment.^A17,A18^ When discussing gender options, clinicians need to consider the legal regulations of the country in which they work as well as the religious and other ideologies that can influence the gender perspectives of patients (and of caregivers for minors). These considerations are also very important when doing clinical work with visitors or immigrants from foreign countries. Thus, the viewpoints of patients (and caregivers) within their cultural contexts should be explored in detail and taken into consideration when these individuals are provided with psychoeducation about gender and other issues related to their intersex conditions.^A19^

As with other transgender patients, when working with patients with an intersex condition and GD, clinicians should engage the patient in a detailed discussion of their expectations from the gender transition: the social effects of public gender change as well as the medical and social effects of the attendant change in hormone treatment and, if desired, of genital or chest surgery. Some of their expectations may be unrealistic, and after detailed discussion, some patients may modify the hormonal and/or surgical treatments they desire or decide against medical treatments or legal gender change, and pursue other ways of finding authenticity in their gender expression. Patients may be happy with their gender-atypical bodies and/or adapt a nonbinary gender identity such as “intersex.” Mental health providers should not assume that patients would benefit from conforming to fit within a gender binary, physically or with respect to gender identity.

Empathic listening is especially important in working with intersex individuals, perhaps particularly with those who have inadvertently discovered their intersex status in adolescence or adulthood, and may have been stigmatized for gender nonconformity or homosexuality, or subjected to irreversible hormonal or surgical treatments consistent with their assigned rather than their experienced gender. Upon discovery of their biological status, such patients may feel betrayed by their parents and physicians, feeling they colluded to keep them in ignorance of their medical condition, damaged their bodies, or punished or stigmatized them for their gendered behaviors. Such patients need empathic validation of their feelings. Assurance that parents and providers had their best intentions at heart, while usually true, is likely to be experienced as an empathic failure and negatively impact the formation of a therapeutic alliance.

As is often seen in many individuals with uncommon medical conditions, many people with intersex conditions experience varying degrees of isolation and loneliness.^A1^ Therefore, linking them to existing intersex support groups by internet or face-to-face meetings can be very beneficial. Despite the emotional relief that support groups can provide, such contacts may sometimes cause additional concerns. For instance, the composition of the group (e.g., the syndromes represented within the group, the personalities of some group members, or the goals of the group) may not meet the individual's expectations, and the information provided may not always be accurate. Thus, some monitoring of the patient's experience with the chosen group is recommended.

### Hormonal and surgical treatments

As reviewed elsewhere,^A1^ many individuals with both an intersex condition and GD will be agonadal in later adolescence or adulthood, either because they were born that way (e.g., in syndromes involving gonadal dysgenesis) or due to surgery, for instance, for the prevention of gonadal malignancy. In those with intact gonads (especially 46,XX congenital adrenal hyperplasia raised female), loss of fertility may be another issue of concern. Persons who are agonadal are usually on hormone replacement therapy by the time of late adolescence. Cessation of that treatment, change to treatment with hormones congruent with their gender identity, patient education for informed consent, and the monitoring of treatment effects are tasks of the endocrinologist.

Also, the technical aspects of genital surgery are more complex than in patients receiving gender-confirming genital surgeries, who do not have intersex conditions. Both the external genitalia and the internal reproductive tract in intersex conditions typically differ from what most surgeons are familiar with in transgender patients without these conditions. In addition, many patients with intersex conditions have already undergone one or more genital surgeries by late adolescence. The resulting postsurgical anatomy constitutes an additional challenge for the surgeon performing gender-confirming surgery, and a good sex-functional outcome may be more difficult to achieve.

Mental health providers should also be aware that not all individuals who identify their gender or gender identity as intersex have a somatic intersex condition, and should ensure that those who do have an intersex condition are receiving adequate medical care, including hormones (to prevent osteoporosis) and cancer screenings, as appropriate to their particular condition.^A3^ Without challenging a patient's identity label, this distinction can usually be made by inquiring about the name of the patient's condition, when and how they learned of it, and any history of related surgeries, hormonal replacement, or ongoing follow-up evaluations. If there is any doubt, appropriate referrals should be made to ensure that the patient is receiving adequate follow-up and treatment.
